# Preparing Medical Students for the Final Examinations During the COVID-19 Crisis: A Bumpy Ride to the Finishing Line

**DOI:** 10.2196/31392

**Published:** 2022-02-03

**Authors:** Nanthini Mageswaran, Noor Akmal Shareela Ismail

**Affiliations:** 1 Faculty of Medicine Universiti Kebangsaan Malaysia Kuala Lumpur Malaysia

**Keywords:** COVID-19, undergraduate medical education, medical students, clinical competency, pandemic

## Abstract

In this viewpoint, we share and reflect on the experiences of final-year students preparing for a high-stakes examination at the Faculty of Medicine, Universiti Kebangsaan Malaysia during the COVID-19 pandemic. We highlight the new challenges faced during web-based remote learning and major differences in the clinical learning environment at our teaching hospital, which was one of the designated COVID-19 centers in Malaysia. We also document how a face-to-face professional examination was conducted for final-year medical students at our institution despite in times of a global health crisis. The lessons learned throughout this process address the importance of resilience and adaptability in unprecedented times. Further, we recommend appropriate measures that could be applied by medical schools across the world to improve the delivery of quality medical education during a crisis in the years to come.

## Introduction

The COVID-19 pandemic has had a profound impact on medical education, particularly in terms of how teaching and assessments are delivered. The immediate effect of the implementation of a nationwide Restriction of Movement Order by the Malaysian government has impacted all students in local medical schools to discontinue educational activities in clinical environments. Our university teaching hospital was declared as COVID-19 designated center in Malaysia to help ease the burden of the national health care system. Clinic sessions, elective medical procedures, and surgeries were all postponed. This resulted in a major shift in the patient pool available at the wards. There were more inpatients with medical or surgical emergencies. Patients suspected of any respiratory infection were admitted to the severe acute respiratory infections wards, limiting the respiratory specialty ward to patients with lung malignancy and chronic respiratory illnesses. The wards were a controlled zone with heightened safety protocols, and staff had to abide by very strict standard operational procedures to prevent the transmission of the virus.

Health care professionals who were involved in undergraduate teaching activities were actively working in the frontlines, providing crucial services to patients with COVID-19 and the community. As a result, it is necessary that medical students have relevant skills and knowledge to respond appropriately if the need arises. The development of pandemic preparedness modules to provide undergraduate students with essential skills has been documented in several studies [1,2]. Another crucial factor to consider is the continuity of medical studies. It is critical to maintain learning continuity as well as a steady supply of doctors and experts for the health care system.

There is a paucity of research on the steps taken by medical schools to ensure academic continuity during a pandemic. Issues such as medical school closure, maintaining safety and hygiene, and leveraging technology for e-learning are frequently prioritized. A recent article published by Sungkyunkwan University School of Medicine discussed a pandemic preparedness module during the Middle East respiratory syndrome outbreak, which involved the formation of a special committee, rescheduling of academic calendar, and conducting clinical clerkships at other institutions. However, the authors pointed out that their module was not holistic enough to cover all areas [3]. The Yong Loo Lin School of Medicine, National University of Singapore documented the use of e-learning tools such as Entrada, which included uploading webcast links and lecture slides, creating web-based quizzes, and setting up and conducting web-based meetings. The authors also highlighted on the modifications made to clinical examination during the COVID-19 pandemic, such as strict safety and hygiene protocols and the use of simulated patients in clinical examination stations [4]. On the other hand, the Duke-National University Singapore Medical School applied the following key principles in organizing final year examinations for medical students, such as strict infection control, cohorting of all participant groups, social distancing of individuals, Zoom-facilitated briefings, and Wi-fi–enabled data-gathering from iPad-based objective structured clinical examination (OSCE) scoring system [5].

In this paper, we highlight the new challenges faced during web-based remote learning and major differences in the clinical learning environment at our teaching hospital, which was one of the COVID-19 designated centers in Malaysia. The lessons learned throughout this process address the importance of resilience and adaptability in unprecedented times.

## Challenges in Medical Education During the Pandemic and its Solutions

During the COVID-19 pandemic, students had to quickly acclimatize to learning totally from home. All classes were transitioned to the internet, with lectures and seminars delivered over virtual platforms such as Zoom and Microsoft Teams. There was a strong sense of appreciation toward clinicians who were on COVID-19 duty, yet still finding the time to teach students. Although the complete switch to web-based learning eased the burden on the faculty and teaching physicians, medical students were facing some challenges while attempting to adapt to the entire process. Among the major challenges faced by students were issues with Wi-Fi connectivity, such as poor internet coverage, low internet speed, and local network congestion. A study has shown that 40% of Universiti Kebangsaan Malaysia (UKM) medical students have a poor internet connection (<5 Mbps) [6]. Some students did not have access to Wi-Fi at their homes owing to low socioeconomic backgrounds. Mobile data packages were just not affordable for these students simply because each web-based lesson would consume a large amount of data. These technical glitches resulted in frequent absenteeism from web-based classes. To overcome this problem, the UKM collaborated with a local corporation to provide free access to SIM cards with mobile data to all students. This was a beneficial program that helped reduce the burden of students, especially for those who depended on mobile data to go through the web-based learning process and also to keep them connected with their lecturers.

Another challenge of e-learning is how to practice clinical skills efficiently [7]. Students were slowly losing touch with their clinical skills while learning from home as there was no exposure to real-life patient encounters throughout the lockdown period. History-taking, physical examinations, and performing simple ward procedures are daily routines for final-year medical students in the clinical setting. Efficient teaching of practical and clinical skills could be overcome by using virtual reality simulators [8]. Being at home, away from the campus environment, the motivation to study was fading away. For educators, the main challenge is to stimulate and sustain the learner’s motivation. One approach to meet this challenge was provided by the Attention, Relevance, Confidence, and Satisfaction (ARCS) model of motivation, which analyzes the motivational characteristics of a group of learners and designs motivational strategies on the basis of this analysis. The ARCS model is based on four motivational concepts: Attention, Relevance, Confidence, and Satisfaction [9]. To overcome the lack of motivation among students, the faculty provided students with access to multiple virtual patient learning environments such as the DxR Clinician software and Medscape Patient Simulations. It is an interactive platform where students could play an active role in managing virtual simulated patients, without the fear of being judged or doubt regarding making a mistake that could be a threat to patient safety, while receiving constructive feedback from the lecturers via the university’s learning management system. This indirectly encouraged students toward learning more rigorously and improving their clinical reasoning skills.

Being at home with no access to patients and their medical records, students were running out of material to present during the virtual case-based discussions. Therefore, these virtual learning platforms came in handy by supplying a pool of problem-based learning case studies created from real patient data. These platforms were a win-win situation for both students and the lecturers. Students were engaged in active, self-directed learning with a variety of patient presentations in the comfort of their homes. In addition, lecturers were able to identify the strengths and weaknesses in student’s clinical reasoning skills and closely monitor their progress via quantifiable assessments and scoring tools in these platforms. All forms of student activities and academic events were conducted virtually, including study groups. One cannot afford to study alone as a final-year medical student because there is a lot to learn in such a short duration. Study groups have encouraged series of discussions and peer teachings to improve knowledge retention. It also acts as a support system for medical students to share their bittersweet experiences in the clinical setting. Owing to the pandemic, study groups had to transition to the internet as well. Case scenarios and mock examination practice questions were discussed via videoconferencing. It is undeniable that nothing could replace the significance of learning from a patient with regard to learning medicine. The real-life patient encounters and lessons we learn from them are aspects that would make the most impact in medical education. However, in a crisis such as the COVID-19 pandemic, flexibility and adaptation are essential to keep the ball rolling.

As months passed by, the COVID-19 situation in Malaysia was only taking a turn for the worse, which then led to multiple extensions to the nationwide lockdown period. Final-year medical students were experiencing anxiety with regard to whether they will have their clinical examinations and graduate on time because of the uncertainties surrounding the COVID-19 pandemic and the hassle of applying for residency training thereafter. Final-year medical students in other parts of the world were being fast-tracked in the final part of their undergraduate journey to increase the frontline workforce on the battle against the COVID-19 pandemic. However, there was a national level policy decision made by the Malaysian Medical Council that medical students in their final year must undertake their exit examination to qualify as a doctor. This examination will test the cognitive and clinical skills of the final year medical students. Hence, medical students were concerned, and doubts were raised about how and when the final examination would take place. Additionally, students were stressed on thinking about how their mental health would fare, given the prospect of months of web-based content and revision, alongside concerns regarding their preparedness for life as a qualified doctor.

After the faculty administration scrutinized all the available options, the decision was to extend the final examination to 1 month after the initially planned date with a similar format to that of the previous years. However, some adaptations were made. First, the timeline was changed. In previous years, UKM medical students were provided a 2-week study leave to prepare for both theory and clinical examinations, and these examinations were conjoined one after the other, without any large remedial period in between. This time around, students were provided a 3-week-long study leave to prepare for the theory assessment. Following that, the faculty provided a 2-month remedial period to better facilitate students to practice clinical skills. Owing to the nationwide lockdown, all medical students have lost a discernible 4 months of clinical exposure. Therefore, students were not entirely sure if the preparations were sufficient for the clinical assessments, which involved interaction with real patients and in conducting medical procedures. However, the remedial period was useful to an extent. Nevertheless, students still faced difficulties in spending time interacting with patients owing to limited access to the wards and patients. Only 6 medical students were allowed to be in the ward at a particular time, and ward rounds were not made compulsory for students to attend. Students were segregated into smaller groups, and rosters were put up at the entrance to every ward. Each small group was allowed to spend limited time in the ward, depending on the respective departments. Year 3 and year 4 medical students also had their clinical rotations concurrently with the final-year students. With so many medical students around and such limited time in the ward, one can only do so much to gain hands-on experience. Being students and the most junior in the medical hierarchy, students are not always fortunate to encounter opportunities to perform procedures while on clinical duty. Students also faced countless rejections from patients as there were simply too many medical students approaching a particular patient on the same day, especially patients with long hospital stays.

The traditional phrase of “see one, do one, teach one” is no longer relevant in this age where medical negligence and litigations are on the rise. Furthermore, a single chance will never be sufficient to attain competence in a particular procedure. This is where simulation provides a great edge. Certain medical schools in Kuala Lumpur, Malaysia, are implementing an open learning concept. The UKM medical faculty has its Basic Simulation Lab (BSL) and Clinical Skills Lab (CSL), where medical students are allowed access at their convenience, to practice procedures while watching related self-instructional videos (SIVs) provided on site [10]. The BSL and CSL were established in collaboration with the Department of Medical Education to facilitate teaching and training in small groups and self-directed learning through modules and videos made available for users. The BSL and CSL are replicas of the actual clinical environment, which provides students with common medical and surgical procedures in accordance with the medical curriculum. It is very suitable as a learning space, especially learning that involves history-taking and physical examination of patients by simulation. Through simulation and mannequins, the learner would have repeated chances to perform specific skills, especially the rare and uncommon ones. Simulations thereby overcome learning merely by chance, which is often insufficient and dangerous in developing the competence of medical personnel [11]. The SIVs are used as a guide for self-practice, and the procedures were recorded and sent to the lecturers for personalized coaching. This method of learning freed up a considerable amount of time for the lecturers and allowed students to practice at their own pace.

Mental health issues were also on the rise during the pandemic. The global prevalence of anxiety and depression among medical students is 33.8% and 33% respectively, both substantially higher than those in the general population [12,13]. Having to take the biggest examination in medical school and also battle through the challenges that come with a global pandemic is mentally taxing. Positive correlations between academic delays and anxiety symptoms have been reported by studies on Chinese college students during the COVID-19 pandemic [14]. Furthermore, every time students approached a new patient, there was always doubt and fear regarding the patient’s COVID-19 status, although they were fully in personal protective equipment. There were no vaccines available in the country yet at that time, and with clinical examinations around the corner, succumbing to COVID-19 and creating a cluster among medical students was the last thing one would expect. Moreover, there was suspicion among students if they will be competent enough to become safe doctors. All these issues were flocking in the form of negative thoughts, which placed students in a constant state of paranoia.

## Planning and Delivery of the Final Examinations

Conducting a high-stakes examination during a pandemic is a challenging and arduous process. Several medical schools have documented their experience of planning and conducting high-stakes examinations during the COVID-19 pandemic [4,5,15]. Multiple sets of web-based facilitated briefings on the flow process and standard operating procedures were conducted before the examinations. Students were required to fill a health declaration form issued by the Ministry of Higher Education to ensure that they were symptom-free, had not been in close contact with patients with COVID-19, and had not travelled overseas for the last 14 days prior to the examination. Strict infection control and personal hygiene measures were taken before entering the examination hall, such as temperature screening, social distancing. and ensuring that every student was wearing a face mask and sanitized their hands. The process and format of the examination were rather similar to those conducted in previous years, except that there was more gap in between tables to ensure proper distancing among candidates.

After a 2-month remedial period, the clinical examinations were conducted. The clinical examinations at the UKM medical school are divided into two components: the long case presentation in the morning followed by the OSCEs in the evening. Most medical students find the clinical examination challenging and nerve-wracking as one cannot predict the case scenarios that would be involved. The examination took place in a special examination ward located in the main building of the teaching hospital. Students were divided into smaller cohorts, and each cohort was managed in a separate circuit with different reporting and holding rooms. Examiners and candidates did not cross over to other circuits. All were required to wear face masks with face shields or goggles and don white plastic aprons, and always maintain social distance throughout the examination. There were no changes made to the format, station design, and content of the examination. As there was some difficulty in recruiting real patients, some stations were replaced with simulated patients. In the long case examination, candidates who were provided case scenarios with simulated patients were only required to take a complete history from the patient and physical examinations were omitted. Hence, the scoring rubric was adjusted accordingly in those stations. The examination coordinators and administration staff eased the transit from station to station by providing students directions. It was a fairly well-organized examination despite the hassle of strict infection control and cleaning in between circuits. Some examiners had access to Wi-Fi–enabled data-gathering for the examination scoring system; however, most of them had to jot down the marks manually on the scoring sheet. To date, there have been no reports of COVID-19 infections among the candidates, examiners, and examination coordinators. The passing rate was similar to that in previous years. Students who failed the final examinations would undergo supplementary clinical rotations for 6 months and have another opportunity to demonstrate their readiness for clinical practice.

## Recommendations and Take-home Messages

The recent COVID-19 pandemic has interrupted the training of medical students worldwide. The lessons learned throughout this process address the importance of resilience and adaptability in all aspects of medical education. Unprecedented times such as this one test the preparedness of the faculty to cater to the learning needs of medical students. Therefore, these are some recommendations that may help improve the quality of medical education among medical students during a pandemic.

### Accessibility to an Internet Connection

The main hindrance to web-based learning during the pandemic was accessibility to an internet connection. As large data are exchanged during web-based classes, a stable, high-speed network is paramount in the campus environment. In lockdown circumstances, where students are learning from home, the university should collaborate with major telecommunication companies to provide students with subsidized mobile data and broadband packages to facilitate remote learning.

### Experimenting With Different Teaching Styles and Learning Methods

Traditional slide-based lectures via virtual platforms could induce boredom, especially when they last long durations. Educators could include games and interactive sections in their lectures to improve student engagement; for example, Kahoot quizzes. Lectures and educational videos could be prerecorded or recorded during the class so that students who have internet connectivity problems during the scheduled class would not fall behind their lessons. In the context of teaching clinical skills, lecturers could invite patients with stable health conditions to join web-based teaching sessions. History-taking and clinical counseling skills, for example, could be carried out with a real patient via videoconferencing instead of student-to-student role plays. In cases where the real patient could not be present, virtual ward rounds could be organized for clinical students. Faculty should also provide students with free access to e-books and scientific journals for convenient referencing. Furthermore, Massive Open Online Courses such as the World Health Organization’s OpenWHO platform should be promoted to students to supplement their learning experience.

### Modifications to Methods of Evaluation and Assessments

There is a need for a fair system of evaluating the academic performances of medical students. During a high-stake examination such as the final-year examinations, all potential barriers faced by medical students should be considered when evaluations are performed. When there is a strict movement control order and state or district borders are closed, selected patients for OSCEs and external examiners would face difficulties in being present on the scheduled examination dates. In special cases of this sort, students could be assessed remotely via videoconferencing for convenience purposes. OSCE stations that require students to be in very close proximity with the patient, such as conducting a fundoscopic examination, should be conducted using appropriate mannequins. Weightage and carry marks should be adjusted accordingly. Long case presentation stations that involve simulated patients and do not require students to perform physical examinations should have a modified marking scheme. In these scenarios, more weightage should be given to the case presentation and quality of discussion in the viva voce session. The final professional examinations are so unique that one only gets to experience it once in their entire medical school life. When circumstances allow, mock examinations should be conducted to familiarize students with the flow process of the examination and also provide them the first-hand feeling of taking part in a high-stakes examination.

## Conclusions

To summarize, we discussed in detail the contingency preparations by the Faculty of Medicine of the UKM, which covered a wide range of aspects such as the curriculum, examination process, as well as safety precautions for all students and staff during the COVID-19 pandemic. In the COVID-19 era, conducting a final-year medical examination poses significant challenges. Hence, medical schools should allow some flexibility when conducting these examinations. We believe that our recommendations will be helpful to other medical schools as they assess their preparedness for a pandemic.

## Abbreviations

ARCS: Attention, Relevance, Confidence, and Satisfaction
BSL: Basic Simulation Lab
CSL: Clinical Skills Lab
OSCE: objective structured clinical examination
SIV: self-instructional video
UKM: Universiti Kebangsaan Malaysia
